# Combined Effects of Rhodiola Rosea and Caffeine Supplementation on Straight Punch Explosive Power in Untrained and Trained Boxing Volunteers: A Synergistic Approach

**DOI:** 10.3390/metabo15040262

**Published:** 2025-04-10

**Authors:** Biaoxu Tao, Hao Sun, Huixin Li, Zhiqin Xu, Yuan Xu, Liqi Chen, Chengzhe Ma, Xiaoyu Zhang, Longqi Yu, Shanjun Bao, Chang Liu

**Affiliations:** 1 School of Sports Training, Wuhan Sports University, Wuhan 430079, China; 2024410681@whsu.edu.cn (B.T.); 2024410703@whsu.edu.cn (H.S.); 2024410712@whsu.edu.cn (X.Z.); 2 School of Sport Science, Beijing Sport University, Beijing 100084, China; 2021011319@bsu.edu.cn (H.L.); 2024210297@bsu.edu.cn (Z.X.); liqi20011004@gmail.com (L.C.); stefanma888@gmail.com (C.M.); 3 The School of Arts, Beijing Sport University, Beijing 100084, China; 2022015616@bsu.edu.cn (Y.X.); yulongqi.johnny@bsu.edu.cn (L.Y.)

**Keywords:** Rhodiola rosea, caffeine, straight punch, muscle explosiveness, muscle endurance

## Abstract

Objectives: This study aimed to investigate the effects of combined supplementation with Rhodiola rosea (RHO) and caffeine (CAF) on the explosive power and sustained output capacity of lead and rear straight punches in both untrained and trained volunteers, with a focus on potential synergistic effects. Methods: randomized, double-blind, placebo-controlled design was employed, enrolling 96 participants (48 untrained, 48 trained). Participants were stratified and randomly assigned to the control (CTR), CAF, RHO, or CAF+RHO group. All subjects completed an 8-week standardized boxing training program (twice per week). Punch performance was assessed using professional boxing equipment and a biomechanical testing system, evaluating lead and rear straight punches, ground reaction force (GRF), and a 30 s continuous punching test. Results: the CAF+RHO  group showed significant improvements in both untrained and trained volunteers. Com-pared to the RHO group, this group demonstrated higher lead punch velocity, shorter bi-lateral peak force time during rear punches, and more punches in the 30 s test (*p* < 0.05). Compared to the CAF group, the CAF+RHO group exhibited greater rear punch force, higher bilateral peak force during lead punches, increased forefoot peak force in rear punches, and improved 30 s power output (*p* < 0.05). The CAF+RHO group also outperformed the CTR group across all parameters (*p* < 0.05). Conclusions: Combined supple mentation with CAF and RHO significantly enhances both explosive power and sustained output in boxing performance. This may result from improved energy metabolism efficiency and neuromuscular coordination, providing a promising nutritional strategy for high-intensity intermittent exercise.

## 1. Introduction

As competitive sports continue to strive for improved performance, the scientific community is increasingly exploring effective strategies to optimize athletic performance and promote physiological adaptations [1,2]. In this context, natural supplements have gained widespread attention due to their potential in enhancing athletic abilities. Among these, Rhodiola rosea (RHO) and caffeine (CAF) have become research hotspots in sports nutrition, due to their significant effects on muscle endurance, explosive power, and delay of fatigue [3].

RHO is a traditional herbal supplement known for its ability to enhance athletic performance with minimal side effects [4]. Its active compounds, rosavin and salidroside, boost anaerobic capacity in type II muscle fibers by activating the skeletal muscle AMPK/Sirt1/PGC-1α signaling axis [5], facilitating mitochondrial biogenesis, and accelerating ATP resynthesis, thereby providing direct energy support for explosive power output [6,7]. Furthermore, RHO stimulates erythropoietin (EPO) expression via the HIF-1 signaling pathway, enhancing sustained oxygen delivery and optimizing muscle contraction efficiency during high-intensity exercise [8].

CAF, as a central nervous system stimulant, enhances performance by antagonizing adenosine receptors [9]. This process promotes the release of neurotransmitters [10], such as dopamine and catecholamines (epinephrine and norepinephrine) [11], increasing skeletal muscle contraction force [12,13]. Moreover, CAF can regulate sympathetic nervous system activity, reduce fatigue, and optimize performance. Its role in promoting calcium ion release further enhances muscle contraction ability [14]. Research has confirmed that moderate CAF (3 mg/kg) intake can enhance anaerobic performance, including muscle explosive power and sustained output [15,16].

**Figure 1:**
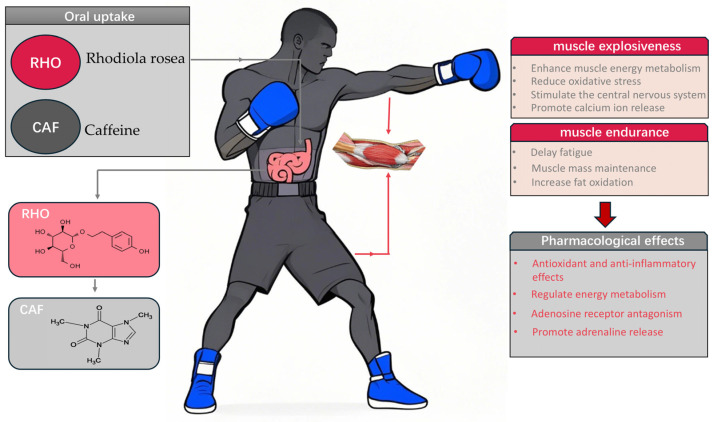
Pharmacological effects of Rhodiola rosea and caffeine, and their roles in promoting muscle explosive power, muscle endurance, and enhancing athletic performance.

Although the individual effects of RHO and CAF have been extensively studied, the synergistic effects of their combined application, particularly in high-intensity intermittent sports like boxing, remain underexplored. Boxing, which demands both explosive power and sustained output [17], serves as an ideal model for investigating the synergistic effects of RHO and CAF. Studies have shown that RHO, through the AMPK/Sirt1/PGC-1α signaling axis [18], plays a critical role in optimizing energy metabolism by regulating mitochondrial function [19]. The active ingredient in RHO, salidroside, activates the Sirt1/PGC-1α axis to enhance mitochondrial biogenesis, with PGC-1α acting as a downstream target of the AMPK pathway [20], directly regulating skeletal muscle oxidative metabolism and fatigue resistance. This suggests that RHO may provide sustained energy support for high-intensity punch output in boxing by enhancing mitochondrial function. Meanwhile, CAF increases central nervous system excitability by blocking adenosine receptors (ADORA1/ADORA2) [21], promoting dopamine release, and enhancing motor unit recruitment efficiency (Figure 1), which in turn improves punch speed and force output. Therefore, exploring whether the combination of RHO and CAF can offer a novel strategy for enhancing boxing performance is crucial [22].

In boxing, a typical explosive power sport, the ability to generate explosive force directly affects the outcome of the match [17]. The 10-point scoring system used in boxing is essentially a comprehensive quantification of an athlete’s punch effectiveness, with the quality of effective punches being the core evaluation indicator [23]. From a biomechanics perspective, the punch effectiveness depends on the product of force and speed, i.e., explosive power. This requires boxers to efficiently coordinate multi-muscle group activities in a very short time to achieve precise force output [24]. Therefore, the strength and speed of punches not only determine the balance between offense and defense but also play a decisive role in gaining an offensive advantage during the match.

Straight punches, as the most fundamental and practical offensive technique in boxing, can be divided into front and rear straight punches [25]. Biomechanically, their characteristics exhibit a “three-point alignment” technical structure: lower-limb drive, trunk rotation acceleration, and upper-limb whip-like release [26]. Compared to curved punch techniques (such as hooks and uppercuts), straight punches have significant kinematic advantages: the shortest movement trajectory, least action time, and highest force transfer efficiency.

Although the final expression of a straight punch is the upper-limb striking motion, its power primarily originates from the ground reaction force (GRF) generated by the lower limbs [23]. According to the kinetic chain theory, the force generated by the lower limbs is transmitted in sequence through the body’s joints, with each segment amplifying the force [27]. Studies have shown that approximately 60–70% of the punching force comes from lower-limb drive, and the kinetic chain mechanism allows boxers to efficiently transfer energy from the ground up through the body, ultimately delivering the maximum force to the fist during the punch [28]. Therefore, evaluating the explosive power of a straight punch requires not only measuring punch speed and impact force but also assessing lower-limb push-off power and force generation speed [29].

This study aims to investigate the potential synergistic effects of CAF and RHO supplementation on the explosive power of straight punches in both untrained and trained boxing volunteers. In addition to supplement intervention, all participants underwent a standardized boxing training program. By using an age-matched human participant model, we aim to determine whether the combined application is superior to the effects of single supplements. Exploring the synergistic effects of CAF and RHO is not only significant for the field of boxing and combat sports but also offers a scientific nutritional supplementation strategy for boxers, promoting the deeper integration of nutrition science and boxing training.

Primary hypothesis: Combined supplementation of CAF and RHO, along with boxing training, will significantly enhance the explosive power of straight punches in both untrained and trained volunteers, compared to the placebo, RHO alone, or CAF alone.

Secondary hypothesis: Combined supplementation will significantly enhance sustained punching capacity, enabling boxers to maintain peak performance during high-intensity punching tasks.

## 2. Materials and Methods

### 2.1. Participants

This study recruited students from Wuhan Sports University and employed an age- and BMI-matched design [30]. A total of 96 volunteers participated, comprising 48 untrained individuals and 48 trained boxers. Both groups were equally stratified into four intervention groups (*n* = 12 per group): control (CTR), caffeine (CAF), Rhodiola rosea (RHO), and caffeine–Rhodiola rosea combination (CAF+RHO) (Table 1). The inclusion criteria for trained volunteers were as follows: (a) 4 years of professional boxing training experience; (b) performing at least two uninterrupted training sessions per week over the past 18 months, each lasting no less than 2 h; (c) a one-repetition maximum (1RM) in the bench press greater than their body weight and a 1RM in the squat greater than 1.5 times their body weight.

Prior to the experiment, researchers provided participants with detailed information about this study’s objectives, procedures, potential benefits, and risks, and obtained written informed consent from all participants. Participants declared that, during the 3 months prior to this study, they adhered to specific health regulations, including not smoking and not having used antidepressants or stimulant drugs. Additionally, each participant completed the Physical Activity Readiness Questionnaire (PAR-Q), with all results indicating no significant health issues and confirming their eligibility for physical activity.

To ensure participants’ health and safety, a comprehensive health assessment was conducted, confirming that all participants had no significant medical history and no history of allergies to Rhodiola rosea or caffeine. Participants were instructed to avoid consuming caffeine-containing foods and beverages and to refrain from vigorous exercise in the 72 h prior to data collection to minimize external influences.

**Figure 2:**
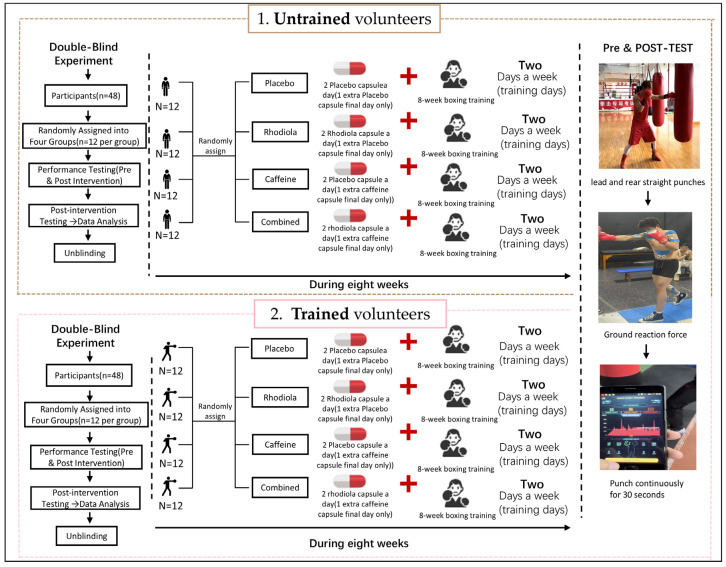
Experimental flowchart.

This study employed a stratified randomization approach for participant allocation. A random sequence was generated using the RANDBETWEEN function in Microsoft Excel, ensuring an even distribution of 48 untrained volunteers into the CTR, CAF, RHO, and CAF+RHO groups, with 12 participants per group (Figure 2). The same procedure was applied to allocate 48 trained volunteers across these groups. To maintain methodological rigor, allocation concealment was strictly enforced, and a double-blind design was implemented to prevent both participants and researchers from knowing group assignments.

**Table 1:** Body characteristics of all participants.

Body Characteristics	Untrained Volunteers (n = 48)	Trained Volunteers (n = 48)
Age (years)	20.38 ± 1.28	22.32 ± 1.18
Height (cm)	176.10 ± 3.87	175.97 ± 4.59
Weight (kg)	74.77 ± 7.43	73.86 ± 4.87
BMI (kg/m^2^)	22.31 ± 1.66	22.17 ± 1.49
Training experience (years)	-	6.63 ± 2.60

### 2.2. Ethics Approval

This study strictly adhered to the principles of the Declaration of Helsinki and was approved by the Ethics Committee of Wuhan Sports University (approval number: 2024121).

### 2.3. Study Design

This study employed an 8-week, randomized, double-blind, placebo-controlled design to assess the effects of supplementation with CAF and RHO on the explosive power of straight punches and GRF in boxers. All groups received boxing training interventions. Testing was conducted two times: pre-test and post-test, following the same procedure for both.

Untrained participants were randomly assigned to one of the four groups (n = 12 per group), while trained participants were similarly randomly assigned to one of the four groups (n = 12 per group) to ensure balanced group allocation. This study utilized a double-blind design, with both participants and researchers blinded to the supplement allocation.

#### The Supplementation Protocol

1. Placebo Group (CTR): To control placebo effects, participants consumed placebo capsules containing starch twice daily, which were identical in appearance to those of the RHO group. On the final test day, to maintain consistency across all groups, participants administered a starch-filled CAF capsule 30 min before testing (Figure 3).

**Figure 3:**
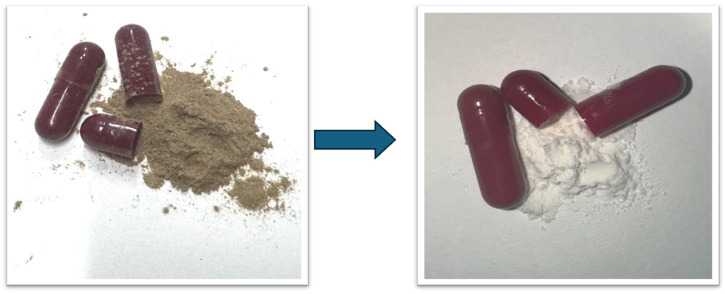
Preparation of placebo.
2. Rhodiola rosea Group (RHO): Participants consumed the RHO extract (2.4 g per day) in two divided doses, which were administered 30 min before breakfast and dinner. On the final test day, to maintain consistency across all groups, participants were administered a starch-filled CAF capsule 30 min before testing.
3. Caffeine Group (CAF): Participants consumed the CAF supplementation (3 mg per kg of body weight) 30 min before the final test day only. During the rest of the intervention period, participants consumed starch-filled RHO capsules to maintain the double-blind design.
4. Combined Group (RHO+CAFs): Throughout the intervention period, participants consumed the RHO extract (2.4 g per day) in two divided doses, administered 30 min before breakfast and dinner. Additionally, they consumed the CAF supplementation (3 mg per kg of body weight) 30 min before the final test day only.

All supplements were distributed by the research team, and the capsules were identical in appearance to maintain the blind procedure. The dosage was determined based on the details and safety guidelines [31].

### 2.4. Supplementation Preparation and Management

CAF was administered using capsules manufactured by Nutricost, based in Utah, USA, with each capsule containing 200 mg of anhydrous CAF, using rice flour and gelatin as excipients. Participants in the CAF Group and CAF+ RHO Group were instructed to take CAF capsules at a dosage of 3 mg per kilogram of body weight. RHO was purchased from Tong Ren Tang Pharmaceutical Company, Beijing, China, with the RHO capsules containing starch and gelatin as excipients. The recommended daily dosage of RHO was 2.48 g [32].

### 2.5. The Training Protocol

To assess the effects of CAF and RHO supplementation on straight punch explosive power and GRF in boxers, an 8-week straight punch training program was designed. The training protocol was based on evidence from Boxing and Combat Sports (ISSN: 1002-7475), along with relevant research from the Journal of Combat Sports and Martial Arts (ISSN: 2084-4301) and the Journal of Sports Sciences (ISSN: 0264-0414)”.

The training regimen included shadow straight punch practice, straight punch on heavy bag, and straight punch target pad training, aiming to develop or refine technical execution while enhancing punching power and speed. Additionally, footwork and reaction training were incorporated to improve lower-limb explosiveness and initiation ability (Table 2). All participants wore heart rate monitors to track real-time fluctuations and adjust training intensity as needed. Under the supervision of professional coaches, participants trained twice per week, with load parameters monitored to ensure safety. Training intensity was progressively adjusted based on the volunteers’ boxing proficiency to prevent overtraining and facilitate adaptation.

**Table 2:** The training program for boxing straight punches.

Exercise	Sets × Reps	Heart Rate Zone	Rest Between Sets
Shadow Straight Punch Practice	4 × 15reps	50–65% Max Heart Rate	1 min
Straight Punch on Heavy Bag	4 × 1 min	60–75% Max Heart Rate	2 min
Straight Punch Target Pad Training	4 × 15reps	50–70% Max Heart Rate	1 min
Footwork Drills	4 × 1 min	50–65% Max Heart Rate	1 min
Reaction Drills (Slip and Straight Punch)	4 × 10reps	60–70% Max Heart Rate	2 min

### 2.6. Daily Dietary Intake Records

Throughout this study, both untrained and trained individuals were required to maintain daily dietary records [32]. To further standardize habitual dietary intake, participants were provided with various packaged food choices that complied with national food safety regulations. Daily dietary intake was monitored during this study, and participants were asked to report their carbohydrate, protein, and fat intake. The total daily energy intake was calculated based on the standard reference from the USDA National Nutrient Database.

### 2.7. The Lead and Rear Straight Punch Test

**Figure 4:**
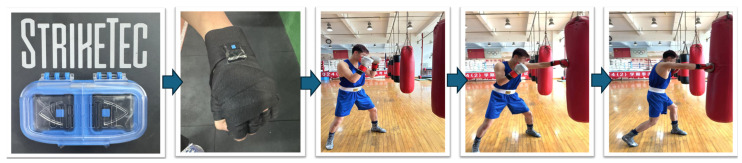
The lead and rear straight punch test.

The lead and rear straight punch test evaluates the explosive power of a boxer’s punches under conditions similar to a boxing match. To assess the explosive power of the front straight punch in both untrained and trained volunteers, participants were grouped by weight. Those in the 52–63 kg group wore 10-ounce boxing gloves, while participants in the 69 kg and above group wore 12-ounce gloves. The STRIKETEC SENSOR KIT was used to measure the force (in pounds) and speed (in miles per hour) of the punches, and the device was securely fixed to each participant’s wrist [33] (Figure 4). Prior to testing, the device was calibrated to ensure it matched each participant’s individual data. The focus of the test was on the force and speed of the front and rear straight punches. Each participant performed three front straight punches and three rear straight punches. Upon hearing the command, participants immediately threw the punches in standard form, with adequate time between each punch to allow for adjustments. The STRIKETEC SENSOR KIT recorded the force and speed of each punch in real time.

### 2.8. The Ground Reaction Force (GRF) Test

Lower-limb initiation power is closely related to punch performance. The ground reaction force (GRF) test is widely used to assess lower-limb strength and speed [34]. During the test, participants stood on a platform with their feet placed one in front of the other, recording the force variation exerted by the legs during the initial movement. The measurements included peak force-to-body weight ratio and peak force time. The peak force-to-body weight ratio was used to assess the relative intensity of the lower-limb force generated during each punch, while peak force time reflected the duration of force application. A one-minute recovery period was observed after each punch. Three valid trials were recorded, and the average value across these trials was used for analysis.

### 2.9. The 30 s Continuous Punching Test

The 30 s continuous punching test is designed to assess the sustained explosive power output over a short duration. Participants stand with their feet staggered and shoulder-width apart, maintaining an upright posture, and making a fist in preparation for rapid punches. At the start of the test, participants punch as quickly as possible, applying maximum force with each punch while maintaining a consistent rhythm. The test lasts for 30 s, during which the total number of punches and the average power output per unit of time are measured and recorded using STRIKETEC SENSOR KIT.

### 2.10. The Motion Analysis System

**Figure 5:**
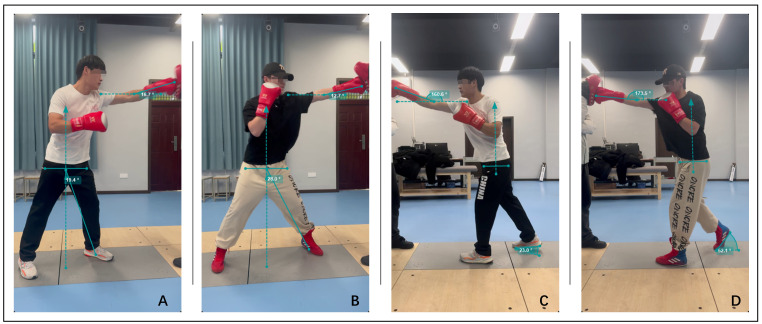
Frames captured in Kinovea: (**A**) Lead straight punch of untrained volunteers. (**B**) Lead straight punch of trained volunteers. (**C**) Rear straight punch of untrained volunteers. (**D**) Rear straight punch of trained volunteers.

Video motion analysis, using software to measure the velocity, acceleration, and dis placement of objects, is widely applied in action analysis. In this study, the Kinovea motion analysis software (version 0.9.5, Kinovea, France) and a standard smartphone were used to record high-speed videos (1920 × 1080 pixels, 60 frames per second) to assist in determining the timing of the punch action based on GRF (Figure 5). The smartphone was mounted on a horizontal tripod at a height of 90 cm for recording. To ensure accuracy in the analysis, the video recording direction had to be perpendicular to the punch action, allowing the software to sample the motion trajectory on the X–Y plane. Additionally, to match the video data to the real-world scale, the software was calibrated using calibration markers. These steps ensured the precision of measurements and the reliability of the analysis [35].

### 2.11. Baseline Data Collection

Baseline data were collected from 48 untrained and 48 trained participants before the intervention to evaluate the initial performance of the four groups (CTR, CAF, RHO, and CAF+RHO) on key indicators. The participants were tested for the following parameters: (1) punch force (N) and speed (m/s) of lead straight and rear straight punches; (2) peak force (N) and time to peak force (ms) of front and rear legs during lead straight and rear straight punches; (3) average punch count (reps) and power output (W) in the 30 s continuous punching test. A one-way ANOVA showed no significant differences among groups in any of these parameters (*p* > 0.05, see Table 3), indicating good baseline homogeneity in performance and providing a reliable reference for evaluating the intervention effects.

### 2.12. Data Statistical Analysis

The results are presented as the mean ± SD. Data from both untrained and trained participants were compared using one-way analysis of variance (ANOVA), followed by Dunnett’s post hoc test for further analysis. The significance level for statistical tests was set at *p* < 0.05 (*), *p* < 0.01 (**) and *p* < 0.001 (***); ns = non-significant. All statistical analyses were performed using Origin 2025 and SPSS 26.0 software [36].

## 3. Results

### 3.1. The Daily Dietary Intake Record

**Figure 6:**
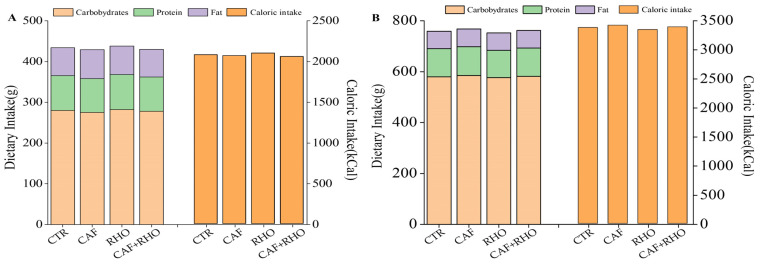
Dietary nutrient intakes and compositions: (**A**) Untrained volunteers. (**B**) Trained volunteers.

To minimize potential confounding effects of dietary variables on study outcomes, standardized dietary interventions were administered to all participants, encompassing both untrained and trained volunteers. Among untrained subjects, the CTR group (2081 kcal), the CAF group (2071 kcal), the RHO group (2102 kcal), and the RHO+CAF group (2060 kcal) maintained high consistency in total caloric intake, with comparable macronutrient distributions of carbohydrates (275–282 g), fat (68–71 g), and protein (83–86 g). Trained volunteers demonstrated a 61.3% average elevation in total energy intake (3348–3422 kcal vs. 2060–2102 kcal in untrained counterparts), primarily attributed to carbohydrate consumption doubling (576–585 g vs. 275–282 g), whereas only moderate variations were observed in fat (68–70 g vs. 68–71 g) and protein intake (108–113 g vs. 83–86 g). The experimental groups for trained participants—CTR (3381 kcal), CAF (3422 kcal), RHO (3348 kcal), and CAF+RHO (3393 kcal)—similarly exhibited stable nutritional patterns characterized by carbohydrates (576–585 g), fat (68–70 g), and protein (108–113 g). These findings indicate that despite minor intergroup discrepancies, no statistically significant differences (*p* > 0.05) were detected in dietary parameters across experimental conditions. As visualized in Figure 6, rigorous adherence to prescribed nutritional protocols by all participants effectively neutralized potential dietary confounding effects on experimental outcomes.

### 3.2. Changes in Untrained Volunteers After Supplementation with Training Supplements

#### 3.2.1. Lead and Rear Straight Punch Force and Velocity

**Figure 7:**
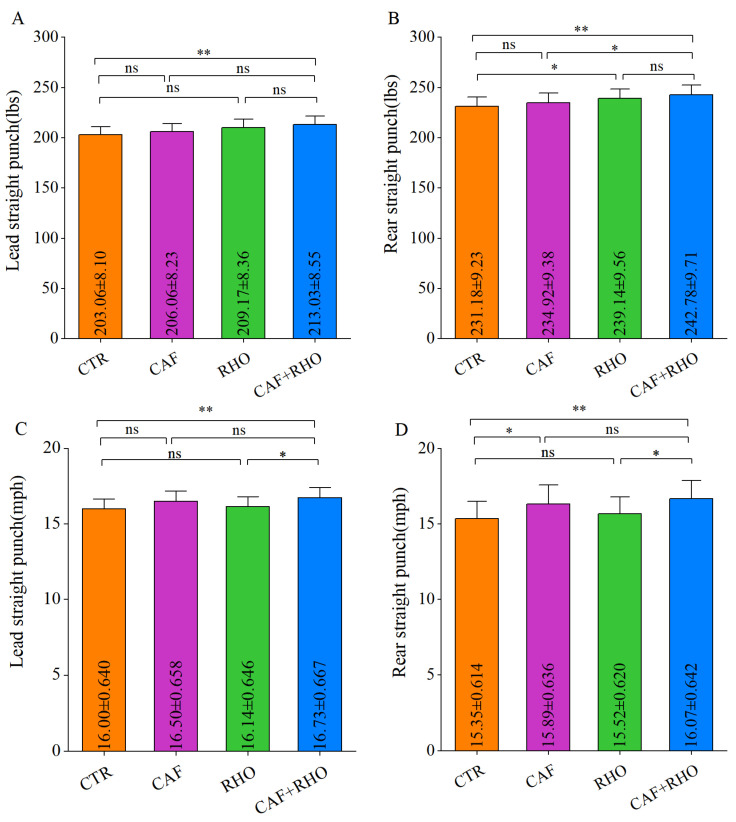
Exercise performance results of untrained volunteers. (**A**) Lead straight punch (lbs). (**B**) Rear straight punch (lbs). (**C**) Lead straight punch (mph). (**D**) R*e*ar straight punch (mph). * *p* < 0.05, ** *p* < 0.01, ns = not significant (*p* ≥ 0.05).

The test results indicated that in the CTR group, the lead straight punch force was 203.06 lbs (Figure 7A) with a speed of 16.00 mph (Figure 7C), while rear straight punch force was 231.18 lbs (Figure 7B) with a speed of 15.35 mph (Figure 7D). The CAF+RHO group demonstrated significant improvements in both force and speed of lead straight and rear straight punches compared to the CTR group (*p* < 0.01). Specifically, for lead straight punch force, the CAF+RHO group was significantly superior to the CTR group (*p* < 0.01), whereas neither the CAF nor RHO group showed significant improvements over the CTR group. In terms of lead straight punch speed, the CAF+RHO group was significantly faster than both the RHO group (*p* < 0.05) and the CTR group (*p* < 0.01). For rear straight punches, the CAF+RHO group exhibited significantly greater punching force than the CAF group (*p* < 0.05) and the CTR group (*p* < 0.01), while punching speed was also significantly higher than in the RHO group (*p* < 0.05) and the CTR group (*p* < 0.01). These findings suggest that, compared to single supplementation with CAF or RHO, their combined supplementation offers superior benefits for improving explosive punching performance.

#### 3.2.2. Peak Force and Peak Force Time in the Ground Reaction Force Test

**Figure 8:**
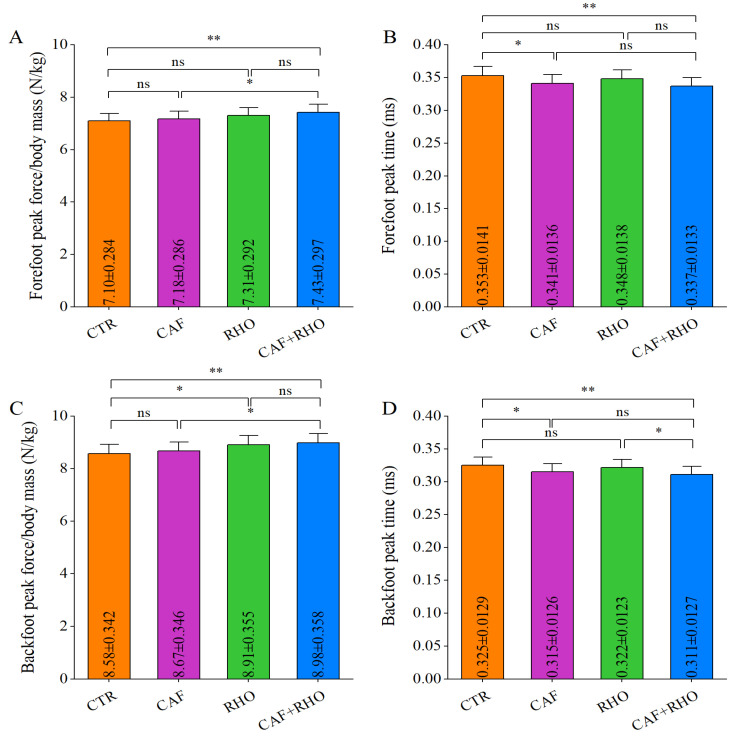
The ground reaction forces of the lead straight punches from untrained volunteers. (**A**) Relative peak force of the forefoot (peak force/body mass). (**B**) Forefoot peak fore time. (**C**) Relative peak force of the backfoot (peak force/body mass). (**D**) Backfoot peak force time. * *p* < 0.05, ** *p* < 0.01, ns = not significant (*p* ≥ 0.05).

GRF is a critical parameter for assessing the lower-limb contribution to punching performance. The GRF test results indicated that in the CTR group, the forefoot peak force during lead straight punches was 7.10 N/kg (Figure 8A), with a peak force time of 0.353 ms (Figure 8B), while the backfoot peak force was 8.58 N/kg (Figure 8C), with a peak force time of 0.325 ms (Figure 8D). The CAF+RHO group exhibited significantly greater peak force and shorter peak force time across all GRF parameters compared to the CTR group (*p* < 0.01). Specifically, in terms of forefoot peak force, the CAF+RHO group was significantly superior to both the CAF group (*p* < 0.05) and the CTR group (*p* < 0.01), whereas neither CAF nor RHO supplementation alone led to significant improvements over the CTR group. Regarding forefoot peak force time, the CAF+RHO group exhibited a significantly shorter duration than both the CTR group (*p* < 0.01) and the CAF group (*p* < 0.05). Similarly, for backfoot peak force, the CAF+RHO group outperformed the CAF group (*p* < 0.05) and the CTR group (*p* < 0.01). Additionally, the CAF+RHO group exhibited a significantly shorter backfoot peak force time compared to the RHO group (*p* < 0.05) and the CTR group (*p* < 0.01).

**Figure 9:**
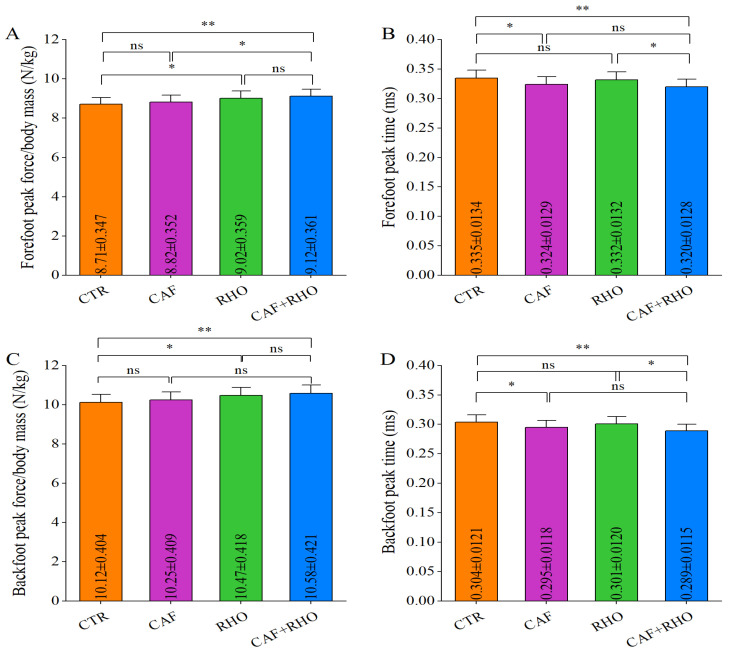
The ground reaction forces of the rear jab punches from untrained volunteers. (**A**) Relative peak force of the forefoot (peak force/body mass). (**B**) Forefoot peak force time. (**C**) Relative peak force of the backfoot (peak force/body mass). (**D**) Backfoot peak force time. * *p* < 0.05, ** *p* < 0.01, ns = not significant (*p* ≥ 0.05).

In the rear straight punch test, the forefoot peak force in the CTR group was 8.71 N/kg (Figure 9A), with a peak force time of 0.335 ms (Figure 9B). The backfoot peak force was 10.12 N/kg (Figure 9C), with a peak force time of 0.304 ms (Figure 9D). The results indicated that the CAF+RHO group exhibited significantly greater forefoot peak force than both the CAF group (*p* < 0.05) and the CTR group (*p* < 0.01), while also demonstrating a significantly shorter forefoot peak force time compared to the RHO group (*p* < 0.05) and the CTR group (*p* < 0.01). In the backfoot test, the CAF+RHO group exhibited significantly greater peak force than the CTR group (*p* < 0.01), along with a significantly shorter peak force time compared to both the RHO group (*p* < 0.05) and the CTR group (*p* < 0.01). Notably, 8 weeks of RHO supplementation and acute CAF intake independently improved lower-limb explosive performance to varying degrees, whereas their combined intervention elicited a significant synergistic effect, optimizing lower-limb force generation.

#### 3.2.3. The 30 s Continuous Punching Test

**Figure 10:**
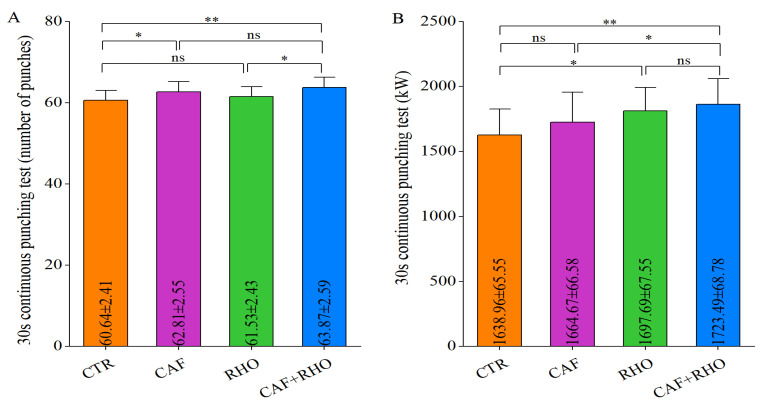
Exercise performance results of untrained volunteers. (**A**) The 30 s continuous punching test (number of punches). (**B**) The 30 s continuous punching test (kW). * *p* < 0.05, ** *p* < 0.01, ns = not significant (*p* ≥ 0.05).

The 30 s continuous punching test was employed to evaluate sustained power output. As illustrated in Figure 10A, the average number of punches in the CTR group was 60.64. Compared to the CTR group, the RHO group failed to show significant improvement, whereas the CAF group exhibited a significant increase (*p* < 0.05). Of particular note, the CAF+RHO group achieved the greatest enhancement (*p* < 0.01), with a significantly greater increase than the RHO group (*p* < 0.05). Regarding average power output (Figure 10B), the baseline value in the CTR group was 1638.96 W. The results indicated that the CAF group did not induce a significant change, whereas the RHO group demonstrated a significant increase (*p* < 0.05). Notably, the CAF+RHO group yielded the most pronounced improvement (*p* < 0.01), with a significantly greater enhancement compared to the CAF group (*p* < 0.05). These findings suggest that the combined supplementation of CAF and RHO enhances short-term explosive power output through a synergistic mechanism, demonstrating superior efficacy over single-supplement strategies.

### 3.3. Changes in Trained Volunteers After Supplementation with Training Supplements

#### 3.3.1.  Change in Lead and Rear Straight Punch Force and Velocity

**Figure 11:**
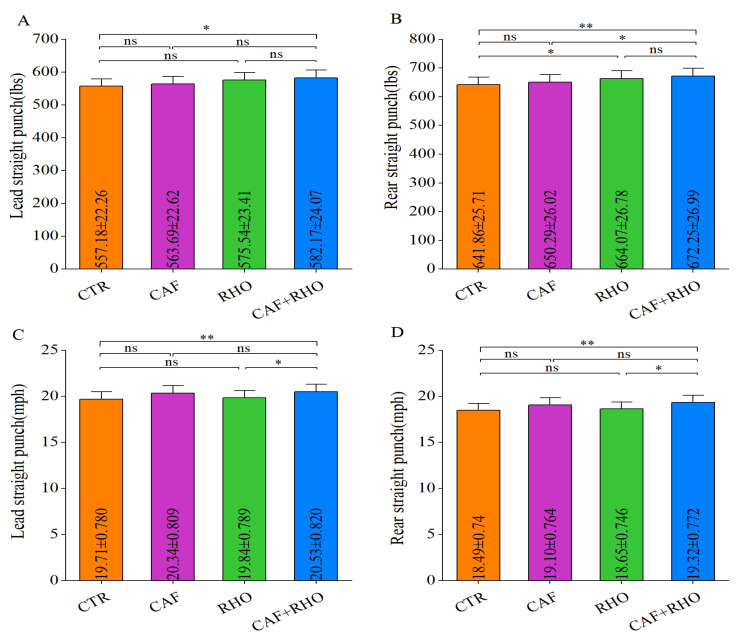
Exercise performance results of trained volunteers. (**A**) Lead straight punch (lbs). (**B**) Rear straight punch (lbs). (**C**) Lead straight punch (mph). (**D**) R*e*ar straight punch (mph). * *p* < 0.05, ** *p* < 0.01, ns = not significant (*p* ≥ 0.05).

The test results indicated that the CTR group had a lead straight punch peak force of 557.18 lbs (Figure 11A) and a punch velocity of 19.71 miles per hour (Figure 11C), while the rear straight punch had a peak force of 641.86 lbs (Figure 11B) and a velocity of 18.49 miles per hour (Figure 11D). Compared to CTR, the CAF+RHO group demonstrated significant improvements across all metrics (*p* < 0.05). Specifically, in the lead straight punch, CAF+RHO group showed significantly greater peak force than CTR group (*p* < 0.05). In the rear punch, RHO group outperformed CTR group in peak force (*p* < 0.05), while CAF+RHO group showed the greatest enhancement (*p* < 0.01), significantly exceeding CAF group (*p* < 0.05).Furthermore, the CAF+RHO group showed significantly higher velocities for both the lead and rear straight punches compared to the RHO and CTR groups (lead: *p* < 0.05; rear: *p* < 0.01). Although the long-term training of the participants may have caused a “ceiling effect”, these findings confirm that the CAF+RHO combined supplementation strategy significantly enhances the explosive power of straight punches in trained athletes, exhibiting a distinct synergistic effect.

#### 3.3.2.  Change in Peak Force and Peak Force Time in the Ground Reaction Force Test

**Figure 12:**
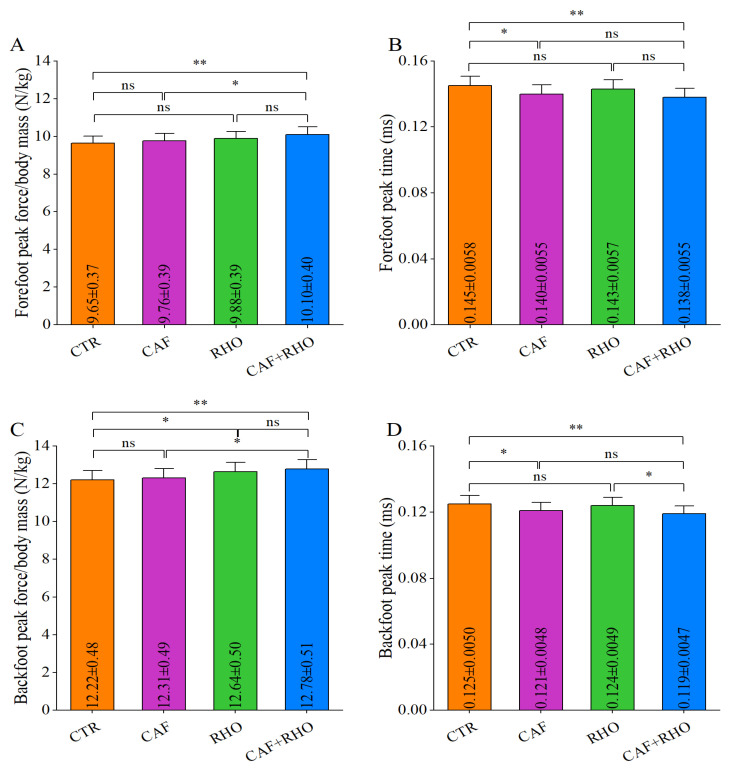
The ground reaction forces of the lead straight punches from trained volunteers. (**A**) Relative peak force of the forefoot (peak force/body mass (**B**) Forefoot peak force time. (**C**) Relative peak force of the backfoot (peak force/body mass). (**D**) Backfoot peak force time. * *p* < 0.05, ** *p* < 0.01, ns = not significant (*p* ≥ 0.05).

The test results showed that in the lead straight punch test, the peak GRF of the front leg in the CTR group was 9.66 N/kg (Figure 12A), with a time to peak of 0.145 ms (Figure 12B), while the rear leg peak force was 12.22 N/kg (Figure 12C), with a time to peak of 0.125 ms (Figure 12D). The CAF+RHO group exhibited significantly greater GRF values across all measured parameters compared to the CTR group (*p* < 0.01). Specifically, the front leg peak force was significantly higher than that of the CAF group (*p* < 0.05) and CTR group (*p* < 0.01), while the time to peak for the front leg was significantly shorter than in the CTR group (*p* < 0.01). Regarding the rear leg peak force, both the RHO group (*p* < 0.05) and CAF+RHO group (*p* < 0.01) showed significant improvements over the CTR group, with the CAF+RHO group also outperforming the CAF group (*p* < 0.05). Furthermore, the time to peak for the rear leg in the CAF+RHO group was significantly reduced compared to the CTR group (*p* < 0.01) and RHO group (*p* < 0.05).

**Figure 13:**
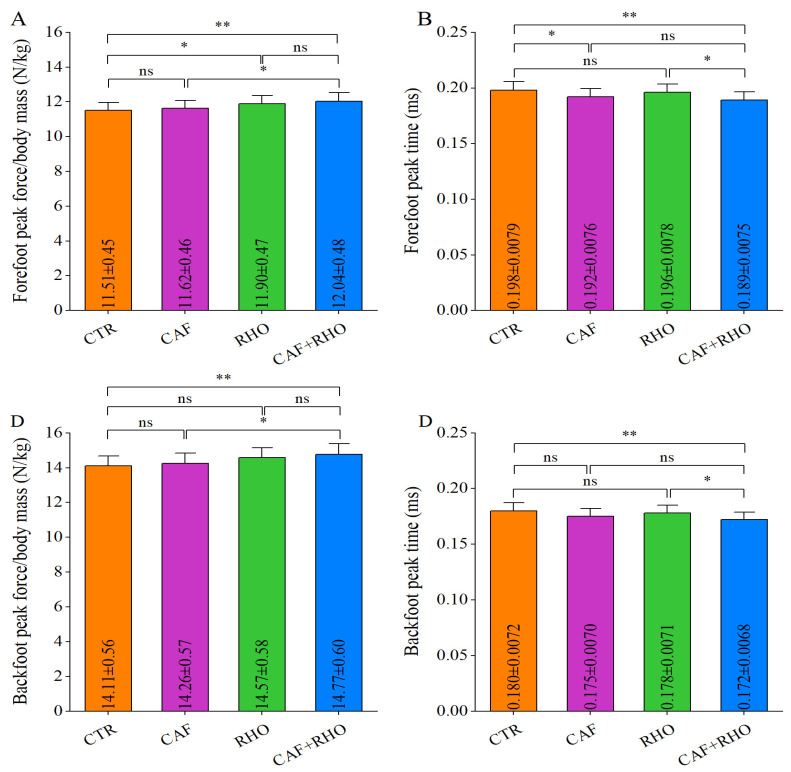
The ground reaction forces of the rear straight punches from trained volunteers. (**A**) Relative peak force of the forefoot (peak force/body mass (**B**) Forefoot peak force time. (**C**) Relative peak force of the backfoot (peak force/body mass). (**D**) Backfoot peak force time. * *p* < 0.05, ** *p* < 0.01, ns = not significant (*p* ≥ 0.05).

In the rear straight punch test, the CTR group recorded a peak force of 11.51 N/kg for the lead leg (Figure 13A), with a time to peak force of 0.198 ms (Figure 13B), and a peak force of 14.11 N/kg for the rear leg (Figure 13C), with a time to peak force of 0.180 ms (Figure 13D). The CAF+RHO group demonstrated significantly higher peak forces for both the lead and rear legs compared to the CAF group (*p* < 0.05) and the CTR group (*p* < 0.01). Moreover, the time to peak force for both the lead and rear legs was significantly shorter in the CAF+RHO group compared to both the RHO group (*p* < 0.05) and the CTR group (*p* < 0.01). These findings confirm that the CAF+RHO combined intervention significantly enhances lower-limb explosive force performance, highlighting a clear synergistic effect.

#### 3.3.3.  Change in the 30 s Continuous Punching Test

**Figure 14:**
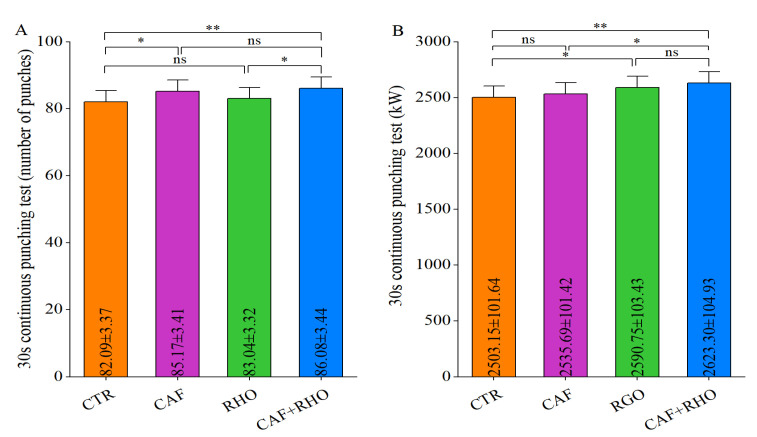
Exercise performance results of trained volunteers. (**A**) The 30 s continuous punching test (number of punches). (**B**) Th*e* 30 s conti*n*uous punching test (kW). * *p* < 0.05, ** *p* < 0.01, ns = not significant (*p* ≥ 0.05).

In the 30 s continuous punching test, the CTR group achieved an average of 82.09 punches (Figure 14A). The results indicated that both the CAF group (*p* < 0.05) and the CAF+RHO group (*p* < 0.01) significantly outperformed the CTR group in terms of punch frequency, with the CAF+RHO group showing superior performance compared to the RHO group (*p* < 0.05). Regarding power output (Figure 14B), both the RHO group (*p* < 0.05) and the CAF+RHO group (*p* < 0.01) exhibited significant improvements over the CTR group, and the CAF+RHO group (*p* < 0.05) also significantly exceeded the CAF group. These results confirm that the combined CAF+RHO intervention produces a synergistic effect, significantly enhancing sustained explosive power performance.

## 4. Discussion

This study aims to assess the effects of CAF and RHO supplementation on straight punch explosiveness, lower-limb peak force, and peak force time in both untrained and trained volunteers. Our results demonstrate that supplementation with CAF and RHO significantly enhanced straight punch explosiveness, as well as lower-limb peak force and peak force time. We rigorously controlled confounding variables to ensure that the observed effects were attributable to the supplementation. Training intensity, experience, diet, and sleep were carefully controlled, meaning that the observed improvements in performance can be attributed to the supplementation, supporting our initial hypothesis [31,32].

The inclusion of both trained and untrained volunteers for RHO supplementation is physiologically significant, particularly due to the high-intensity interval nature of boxing, which alternates between periods of high and low intensity [37,38]. This study demonstrates that RHO supplementation enhances strength, fatigue resistance, and sustains explosive power output, aligning with previous research findings [39,40,41]. RHO improves force production by promoting fatty acid β-oxidation, activating the PGC-1α/NRF1 pathway, facilitating mitochondrial biogenesis, and enhancing ATP generation. Additionally, RHO can co-activate the erythropoietin (EPO) pathway, promoting hemoglobin synthesis and thereby enhancing endurance capacity [42,43]. Additionally, RHO significantly enhances continuous explosive power output through a dual-modulation mechanism. At the physiological regulation level, its active ingredient, salidroside, stabilizes heart rate and optimizes cardiac output during exercise [42]. In terms of antioxidant defense, the levels of oxidative stress markers in skeletal muscle play a critical role in muscle function regulation. Research has shown that RHO effectively inhibits oxidative stress and significantly increases oxygen consumption in muscle fibers [44], an important indicator of mitochondrial respiratory activity in skeletal muscle [45]. This suggests enhanced mitochondrial oxidative phosphorylation, which plays a key role in improving exercise performance [46,47].

RHO’s effects on exercise-induced fatigue are multifaceted, addressing multiple aspects of performance [42]. Studies have shown that 30 days of RHO supplementation before exercise alleviates fatigue by increasing glycogen content, upregulating lipogenesis, and activating protective mechanisms [48]. During exercise, RHO inhibits the abnormal release of creatine kinase (CK) and lactate dehydrogenase (LDH) caused by intense exercise, while reducing C-reactive protein (CRP) inflammation levels [39]. After exercise, RHO supplementation reduces lactate accumulation, inhibits the release of CK, and modulates changes in C-reactive protein [49]. Animal experiments have confirmed that RHO significantly alleviates muscle tissue damage caused by intense exercise in rats and improves the levels of fatigue-related biomarkers, such as lactate dehydrogenase and creatine kinase [50]. Clinical studies further demonstrate that RHO supplementation reduces fatigue and muscle damage after exercise, significantly enhancing performance [51]. In this study, the RHO group showed greater punching force in both the front and rear straight punch tests, while also significantly enhancing power output in the 30 s continuous punching test, confirming its potential to improve strength, sustain performance, and delay fatigue.

The positive effects of CAF supplementation on explosive power have been extensively documented. Studies have shown that caffeine intake can significantly enhance athletic speed and power output in combat sports athletes [52]. These effects can be understood through several physiological mechanisms: Firstly, in neuromuscular regulation, CAF antagonizes the A1/A2 adenosine receptors (ADORA1/ADORA2), which increases dopaminergic neuron activity and cortical excitability [53]. This results in improved motor unit recruitment and a reduction in muscle soreness and perceived force [16,54]. Moreover, CAF promotes the opening of the ryanodine receptor 1 (RyR1) channels in the sarcoplasmic reticulum, leading to greater Ca^2+^ transients and an increased rate of cross-bridge cycling [55]. Additionally, CAF partially activates the Na^+^/K^+^ pump, enhancing excitation-contraction coupling and stabilizing action potential conduction [3]. On the metabolic level, pre-exercise plasma FFA concentrations increase significantly. CAF suppresses phosphofructokinase (PFK) activity, reducing glycolysis rates and triggering a glycogen-sparing effect [14,56]. Although the accumulation of anaerobic metabolites decreases the glycolytic rate, the increased Ca^2+^ sensitivity in type II muscle fibers postpones peak blood lactate accumulation [57]. In this study, the CAF group exhibited greater velocity in the front and rear straight punches, GRF, and 30 s punching test, demonstrating its efficacy in enhancing explosive power.

In sports nutrition, when the combination of two components is mechanistically justified and proves more effective than individual use, this is sufficient to establish a synergistic effect [58]. Notably, in this study, both untrained and trained volunteers in the CAF+RHO group exhibited the best performance in multiple tests, with the most significant improvement observed in the 30 s continuous punching test, supporting the presence of a synergistic effect between these supplements. The combined effect of CAF and RHO can be explained through mechanisms involving metabolic optimization, neuromodulation, and enhanced mitochondrial function [59]. RHO supports muscle contraction by promoting fatty acid oxidation and ATP production [41,60,61], while CAF enhances central nervous system (CNS) excitability, boosting motor unit firing frequency and improving force output efficiency. Together, they complement each other by optimizing energy supply and neural drive. Furthermore, the combination of CAF and RHO synergistically activates autophagy in skeletal and cardiac muscles, removing dysfunctional mitochondria and promoting mitochondrial biogenesis [62]. This significantly enhances oxygen consumption and oxidative phosphorylation activity in muscle fibers, thereby optimizing overall energy metabolism efficiency. Finally, RHO regulates mitochondrial biogenesis through the AMPK/Sirt1/PGC-1α pathway [63], while CAF works by antagonizing adenosine receptors to enhance neural excitability [32]. These two mechanisms work synergistically at the molecular level to improve performance. In summary, compared to single supplementation, the combined effect of CAF and RHO significantly enhanced physical performance, demonstrating their strong synergistic potential.

In the front and rear straight punch tests, both untrained and trained volunteers in the CAF+RHO group demonstrated the most significant improvements in punching force and speed. Compared with the CTR group, the explosive power of the CAF+RHO group was significantly enhanced, with differences reaching statistical significance (*p* < 0.01 and *p* < 0.05, respectively), indicating that the combined supplementation of CAF and RHO may effectively boost explosive power output Furthermore, the GRF during the front and rear straight punches in the CAF+RHO group was also significantly higher than that in the CTR group (*p* < 0.01 and *p* < 0.05, respectively) further confirming the enhancement of explosive power. This result aligns with previous studies, suggesting that CAF supplementation can improve athletic performance by increasing motor unit firing frequency and force output efficiency [64,65]. The addition of RHO may further amplify this effect, allowing volunteers, regardless of whether they have received boxing training, to maintain a higher performance level in boxing [61,66].

It is worth emphasizing that the training program employed in this study was based on a classic boxing training regimen [67], which included the front straight punch, rear straight punch, and footwork movements. This training program incorporates enhancements in strength, speed, footwork, and reaction time, serving as a crucial factor in the technical refinement of the straight punch [68].

Trained boxing volunteers, whose performance nears physiological limits, exhibit a significant “ceiling effect”, making single interventions or supplements less effective in yielding noticeable improvements. However, the combined supplementation of CAF and RHO, through synergistic effects, can still extend physiological boundaries, leading to marginal improvements in performance.

However, a key limitation of this study is that it did not include training on other boxing techniques, such as the hook and uppercut, which may have introduced minor variability into the results. Future research should aim to enhance the accuracy of evaluating the effects of CAF and RHO supplementation on boxing performance by systematically incorporating training across a wider range of boxing techniques. This approach will help to reveal the multifaceted effects of supplementation in boxing training more comprehensively, thus providing more reliable evidence for the optimization of sports nutrition strategies.

However, this study has certain limitations. First, the relatively small sample size, which is limited to both untrained and trained volunteers, may restrict the generalizability of the results. Future studies should consider expanding the sample size to include athletes from various genders, sports disciplines, and competitive levels to enhance the general applicability of the findings. Additionally, the lack of measurement of critical physiological and biochemical markers, such as blood glucose, blood lactate, or PI3K-Akt pathway-related markers targeted by miR-486-5p, limits our ability to fully explore the specific mechanisms of action of the supplements. Future studies should employ multi-omics analyses to gain deeper insight into the molecular mechanisms of CAF and RHO supplementation.

## 5. Conclusions

This study suggests that 8 weeks supplementation of RHO combined with a single dose of CAF can significantly enhance the explosive power of straight punches and improve sustained output ability in both untrained and trained individuals. This finding provides a new nutritional strategy for combat sports to optimize athletic performance during high-intensity training and competition.

## Figures and Tables

**Table 3:** Baseline values.

Training Status	Type of Punch	Group	Punching Force (lbs)	Punching Speed (mph)	Peak Force of Front Leg (N/kg)	Peak Force Time of Front Leg (ms)	Peak Force of Rear Leg (N/kg)	Peak Force Time of Rear Leg (ms)	Number of Punches	kW
Untrained Volunteers	Lead Straight Punch	CTR	161.06 ± 6.41	14.14 ± 0.564	6.32 ± 0.252	0.390 ± 0.0156	4.18 ± 0.167	0.366 ± 0.0145	-	-
		CAF	162.43 ± 6.50	14.16 ± 0.565	6.34 ± 0.253	0.386 ± 0.0152	4.24 ± 0.170	0.362 ± 0.0144	-	-
		RHO	157.45 ± 6.30	14.12 ± 0.561	6.27 ± 0.249	0.393 ± 0.0157	4.14 ± 0.165	0.371 ± 0.0146	-	-
		CAF+RHO	159.28 ± 6.36	14.14 ± 0.563	6.29 ± 0.251	0.391 ± 0.0155	4.16 ± 0.166	0.370 ± 0.0148	-	-
	Rear Straight Punch	CTR	183.07 ± 7.33	13.19 ± 0.526	7.55 ± 0.302	0.383 ± 0.0153	4.27 ± 0.170	0.350 ± 0.0140	-	-
		CAF	185.46 ± 7.41	13.22 ± 0.529	7.61 ± 0.304	0.378 ± 0.0150	4.35 ± 0.174	0.347 ± 0.0138	-	-
		RHO	182.56 ± 7.30	13.18 ± 0.525	7.42 ± 0.297	0.387 ± 0.0154	4.16 ± 0.15	0.354 ± 0.0142	-	-
		CAF+RHO	184.62 ± 7.37	13.19 ± 0.528	7.53 ± 0.301	0.384 ± 0.0151	4.22 ± 0.167	0.352 ± 0.0139	-	-
	30 s Continuous Punching Test	CTR	-	-	-	-	-	-	57.61 ± 2.29	1590.72 ± 63.58
		CAF	-	-	-	-	-	-	56.34 ± 2.24	1611.92 ± 64.46
		RHO	-	-	-	-	-	-	56.93 ± 2.27	1567.23 ± 62.78
		CAF+RHO	-	-	-	-	-	-	56.43 ± 2.25	1530.21 ± 61.18
Trained Volunteers	Lead Straight Punch	CTR	557.18 ± 22.26	19.71 ± 0.780	9.65 ± 0.37	0.145 ± 0.0058	12.22 ± 0.48	0.125 ± 0.0050	-	-
		CAF	563.69 ± 22.62	20.34 ± 0.809	9.76 ± 0.39	0.140 ± 0.0055	12.31 ± 0.49	0.121 ± 0.0048	-	-
		RHO	575.54 ± 23.41	19.84 ± 0.789	9.88 ± 0.39	0.143 ± 0.0057	12.64 ± 0.50	0.124 ± 0.0049		
		CAF+RHO	582.17 ± 24.07	20.53 ± 0.820	10.10 ± 0.40	0.138 ± 0.0055	12.78 ± 0.51	0.119 ± 0.0047		
	Rear Straight Punch	CTR	641.86 ± 25.71	18.49 ± 0.740	11.51 + 0.45	0.198 + 0.0079	14.11 ± 0.56	0.180 + 0.0072	-	-
		CAF	650.29 ± 26.02	19.10 ± 0.764	11.62 + 0.46	0.192 + 0.0076	14.26 ± 0.57	0.175 + 0.0070		
		RHO	664.07 ± 26.78	18.65 ± 0.746	11.90 + 0.47	0.196 + 0.0078	14.57 ± 0.58	0.178 + 0.0071	-	-
		CAF+RHO	672.25 ± 26.99	19.32 ± 0.772	12.04 + 0.48	0.189 ± 0.0075	14.77 ± 0.60	0.172 + 0.0068		
	30 s Continuous Punching Test	CTR	-	-	-	-	-	-	82.09 ± 3.37	2503.15 ± 101.64
		CAF							85.17 ± 3.41	2535.69 ± 101.42
		RHO	-	-	-	-	-	-	83.04 ± 3.32	2590.75 ± 103.43
		CAF+RHO							86.08 ± 3.44	2623.30 ± 104.93

## Data Availability

The original contributions presented in this study are included in this article. Further inquiries can be directed to the corresponding authors.
